# PPARβ/δ: Benefits in Coronary Artery Disease and Beyond

**DOI:** 10.5935/abc.20190228

**Published:** 2019-12

**Authors:** Viviane O. Leal

**Affiliations:** Universidade Federal Fluminense, Niterói, RJ - Brazil

**Keywords:** Coronary Artery Disease, Oxidative Strss, Inflammation, Obesity, Hypertension, Dyslipidemias, Risk Factors/prevalence, Myocardial Infarction, Heart Failure.

Peroxisome proliferator-activated receptors (PPARs) are nuclear receptors that participate in nutrient and energy metabolism.^1^ In a recent paper entitled “Nrf2, NF-κB and PPAR β/δ mRNA expression profile in patients with coronary artery disease” (CAD), Barbosa et al. found that PPARβ/δ was highest expressed in the CAD patients when compared to patients without CAD.^2^ Beyond its heart-protective effects associated to improvement of cardiac function and amelioration, the pathological progression of cardiac hypertrophy, heart failure, cardiac oxidative damage, ischemia-reperfusion injury, lipotoxic cardiac disfunction and lipid-induced cardiac inflammation,^3^ others functions PPARβ/δ deserve be considered in the wide context of the cardiovascular disorders.

Obesity and dyslipidemia are risk factors for cardiovascular disease^4^ and, in this sense, the modulation of PPARβ/δ can be interesting because it is associated with the improvement of fatty acid (FA) catabolism in skeletal muscle or alternating fibre type muscle during oxidative metabolism.^1,5^ PPARβ/δ activation also reduces pre-adipocyte proliferation and differentiation, and attenuates angiotensin II-mediated dysfunctional hypertrophic adipogenesis and inhibits inflammation in adipose tissue.^5^ Besides that, in the intestine, PPARβ/δ can induce the production of short-chain fatty acid (SCFA) production^1^ and butyrate and propionate, two SCFA, were associated with reduction in food intake.^6^ Moreover, PPARβ/δ improves hepatic FA oxidation which decreases the lipids availability for triglycerides synthesis and changes the expression of several apoproteins,^5^ contributing for elevating plasma levels of high-density lipoprotein and decline levels of low-density lipoprotein.^1^

Thus, PPARβ/δ can be a potential target in metabolic disorders.^5^ So, a question is pertinent: how to modulate PPARβ/δ? In the grouP of natural ligands, this subtype is activated by carbaprostacyclin, components of very low-density lipoprotein and unsaturated FAs.^7^

Unfortunately, PPARβ/δ has not been so intensely studied like the subtypes α and γ^7^ and little is known about the potential natural activators, even in the case of unsaturated FAs that can be easily obtained by diet and supplements. So, let's look forward to this answer: it is possible to modulate PPARβ/δ by dietetic bioactive compounds? Nonpharmacologic strategies to modulate other nuclear factors, such as nuclear factor erythroid 2-related factor 2 (Nrf2), been pointed^8^ and it is wanted to the same with PPARβ/δ. Caffeine,^9^ genistein^10^ and non-occidental diet pattern^11^ already look promising.
